# The experimental investigation of foot slip-turning motion of the musculoskeletal robot on toe joints

**DOI:** 10.3389/frobt.2023.1187297

**Published:** 2023-08-29

**Authors:** Kawinna Nipatphonsakun, Takumi Kawasetsu, Koh Hosoda

**Affiliations:** Department of Systems Innovation, Graduate School of Engineering Science, Osaka University, Osaka, Japan

**Keywords:** musculoskeletal robot, robot locomotion, slip-turning, toe joint, foot structure, foot contact area

## Abstract

Owing to their complex structural design and control system, musculoskeletal robots struggle to execute complicated tasks such as turning with their limited range of motion. This study investigates the utilization of passive toe joints in the foot slip-turning motion of a musculoskeletal robot to turn on its toes with minimum movements to reach the desired angle while increasing the turning angle and its range of mobility. The different conditions of plantar intrinsic muscles (PIM) were also studied in the experiment to investigate the effect of actively controlling the stiffness of toe joints. The results show that the usage of toe joints reduced frictional torque and improved rotational angle. Meanwhile, the results of the toe-lifting angle show that the usage of PIM could contribute to preventing over-dorsiflexion of toes and possibly improving postural stability. Lastly, the results of ground reaction force show that the foot with different stiffness can affect the curve pattern. These findings contribute to the implementations of biological features and utilize them in bipedal robots to simplify their motions, and improve adaptability, regardless of their complex structure.

## 1 Introduction

Musculoskeletal robots are designed to imitate the structure and movement of actual beings (Hosoda et al., 2008; Narioka and Hosoda, 2011; Asano et al., 2012) to utilize their biological features and realize more natural motion or human-like motion in humanoid robots (Nakanishi et al., 2013; Kurumaya et al., 2016; Asano et al., 2019). However, one of the main challenges facing these robots is their limited range of motion due to the lack of degrees of freedom (DoFs) (Ogawa et al., 2011), caused by minimizing the robot design to reduce complications of the controller, which can affect their ability to perform both static and dynamic tasks that require complex controls and coordination of muscles. Turning motion is one of those complicated motions that require substantial effort and multiple steps to execute. Furthermore, turning motion is a maneuver that often requires precise control and a wide range of movements, which are the limitations of musculoskeletal robots. To overcome these limitations and improve the functionality of bipedal robots, researchers are focusing on developing new techniques and methods for enhancing their ability to perform motion, such as slip-turning motion.

Slip-turning is a turning mechanic that is said to overcome the physical limitations of robots (Yeon and Park, 2014). Previous studies have shown that slip-turning is more energy-efficient than traditional step turning methods (Hashimoto et al., 2011). Together with the utilization of toe joints, the robots could reach higher mobility, allowing for effective and quick slip-turning using the toes as contact points (Miura et al., 2013). These studies have demonstrated that utilizing these toe joints enables the robots to perform slip-turning motions by minimizing friction-induced power generation and maintaining foot contact on a small support area for a short duration (Miura et al., 2012). Meanwhile, the plantar intrinsic muscle (PIM), which connects to the toes underneath the foot, plays a role in stiffening the toe joints, preventing them from over-dorsiflex or floating toes, possibly leading to improved postural stability and balance during an unstable state (Ku et al., 2012; Ferrari et al., 2020; Fujimaki et al., 2021). Additionally, the stiffness of the PIM contributes to the ability to perform quick motions (Yuasa et al., 2018) and enhance the push-off force (Farris et al., 2019; Farris et al., 2020).

In this paper, we propose an experimental investigation of the foot slip-turning motion of the musculoskeletal robot equipped with toe joints and PIM. The objective is to improve the musculoskeletal robot’s slip-turning capabilities. According to our hypothesis, implementing slip-turning on toe joints in a musculoskeletal robot is expected to yield increased body rotational angle, regardless of whether there are joints in the yaw axis or the vertical axis of the human. Additionally, we propose that the activation of the PIM during motion can affect foot stiffness, which can influence the robot’s postural stability and capability to perform quick movements.

A series of experiments were conducted to assess the effectiveness of toe joints in enabling foot slip-turning. The first experiment aimed to compare slip-turning performance between a foot equipped with toe joints and a foot without toe joints. The second experiment focused on demonstrating the role of the PIM in preventing over-dorsiflexion of the toes, thus improving the robot’s postural stability. In the final experiment, the active utilization of the PIM was investigated to determine its impact on motion propulsion.

The findings of this study could contribute to the advancement of musculoskeletal robot locomotion by introducing a novel approach using the foot structure to improve slip-turning capabilities. The flexibility of the joints combined with the active control of the PIM could provide a versatile solution for achieving agile and efficient movements, enhancing the robot’s adaptability in various real-world scenarios. Future research can focus on further optimizing the design and control of the toe joints, exploring their potential applications in more complex locomotion tasks, and investigating the integration of similar mechanisms in other robotic systems or implementations for medical purposes such as gait training or rehabilitation.

The remainder of this paper is organized as follows: Section 2 provides the structural design of the musculoskeletal robot as in hardware design, pneumatic actuators, and its control system, including the range of motion of the constructed robot. Section 3 explains the slip-turning strategies and the generation of the muscle activation pattern to be used in the experiments. Section 4 describes the experimental setup and methodology of using various foot conditions for evaluating the slip-turning performance. Section 5 presents the results and analysis of the experiments, followed by a discussion of the findings in Section 6. Finally, Section 7 concludes the paper, highlighting the contributions of this research and outlining future directions for investigation.

## 2 Design of musculoskeletal robot with toe joints

### 2.1 Hardware design

For imitation of the movements of biological beings, applying the musculoskeletal structure to a robot is a viable approach to mimic the biomechanical characteristics of living systems. This approach allows robots to replicate the biological features that motor-driven robots cannot achieve with their mechanical design alone. On the other hand, the entire musculoskeletal structure could be challenging to implement in a robot due to its complexity and sophistication. To address this problem, a simplified version of the design is adopted, focusing on the key components necessary for realizing the desired robot motion to alleviate the complexities associated with control architecture.

The number of muscles required for the robot’s turning motion was chosen based on the biomechanical studies on human turning gait (Hase and Stein, 1999) and muscle synergy studies related to human turning mechanics (Ventura et al., 2015; Choi et al., 2017). From these studies, we identified six key muscles that exhibited significant activations during the observation: erector spinae, gluteus medius, vastus lateralis, biceps femoris, soleus, and tibialis anterior. Since the gluteus medius has a complex fan-shaped structure and serves as both the flexor and extensor of the hip, we decided to include an additional muscle, the iliopsoas, to specifically act as the flexor muscle of the hip in our robot. As our robot focuses on the lower half of the body, the erector spinae muscle was excluded. In addition to these muscles, our research also explores the utilization of the foot structure, including toe joints and the PIM, as both features were added to the design of our robot. The placement of joints was carefully done along the pitch axis (or the lateral axis of the human body) to ensure a sufficient range of motions for recreating the swing motion of both legs in the front and back direction, which is considered enough to realize slip-turning in this study.

**FIGURE 1:**
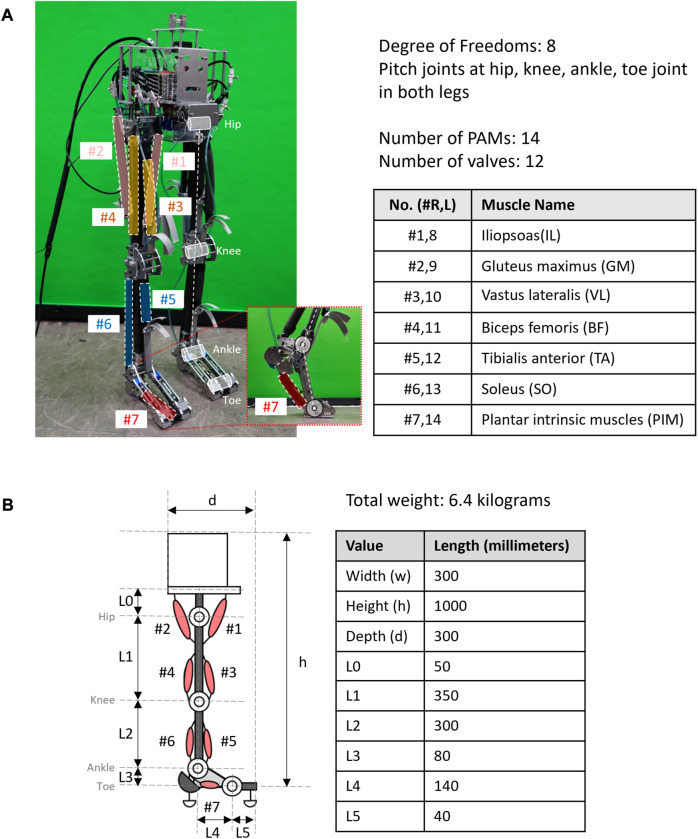
Structural design of the musculoskeletal robot “PneuTurn‐T.” **(A)** The musculoskeletal robot has a total of eight DoFs, four joints in each leg at the hips, knees, ankles, and toes. Each leg has seven PAMs to actuate and restrict the motion of joints, controlled by 12 valves. **(B)** Size and dimension of the bipedal robot.

As a result, the structure of the bipedal robot consists of four joints and seven muscles in each leg, eight DoFs in total. Figure 1B illustrates the leg configuration, which includes a hip joint, a knee joint, an ankle joint, and a toe joint, all placed in the pitch axis. These joints are connected to specific muscles: the gluteus maximus (GM) and iliopsoas (IL) actuate the hip joint, the biceps femoris (BF) and vastus lateralis (VL) actuate the knee joint, the soleus (SO) and tibialis anterior (TA) actuate the ankle joint, and finally, the plantar intrinsic muscle (PIM) actuates the toe joint.

The bipedal musculoskeletal robot named “PneuTurn‐T” (see Figure 1A) was developed for experimental purposes in this study. The robot has a dimension of 300 mm × 1,000 mm × 300 mm (W × H × D), with a weight of 6.4 kg. During the experiments, the robot was connected to an external power supply and air supply to operate.

### 2.2 Pneumatic actuators and controller

**FIGURE 2:**
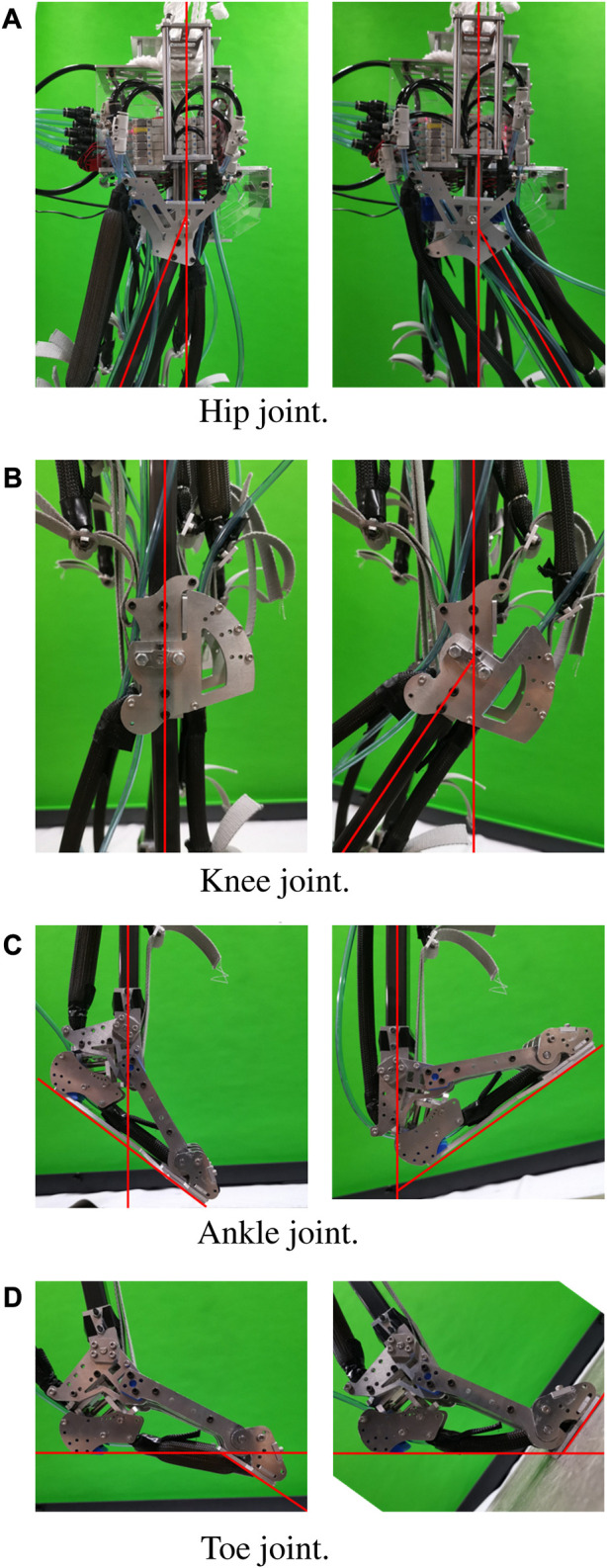
The range of motion at each joint of the musculoskeletal robot is restricted by its structure. **(A)** Hip joints range from −20° to 30°. **(B)** Knee joints range from −45° to 0°. **(C)** Ankle joints range from −30° to 45°. **(D)** Toe joints range from −35° to 60°.

The musculoskeletal robot is driven by pneumatic actuators called pneumatic artificial muscles (PAMs) or the McKibben artificial muscles. The PAMs are capable of acting as muscles of the biological being by contracting and stretching themselves, pulling tendons, and driving the joints. Pneumatic actuators offer some advantages over hydraulic or electric actuators, other than their elasticity. They provide a simple and cost-effective solution with a high power-to-weight ratio, making them suitable for building light-weighted robots. The PAMs offer quick and responsive operation, allowing for rapid changes in direction with a wide range of motions, and can be easily replaced to change the parameter. Our McKibben muscles are made of an 8-mm-diameter rubber tube with 1 mm thickness, with both ends plugged using pneumatic fittings; one closed-end and one open-end connected to an air tube and covered with polyester braided sleeves. The length of each PAM and its contraction ratio is calculated before the fabrication of the PAMs and then measured and tested before being implemented in the robot to accomplish the desired range of motion, referring to the average human range of motion (see Figure 2).

**FIGURE 3:**
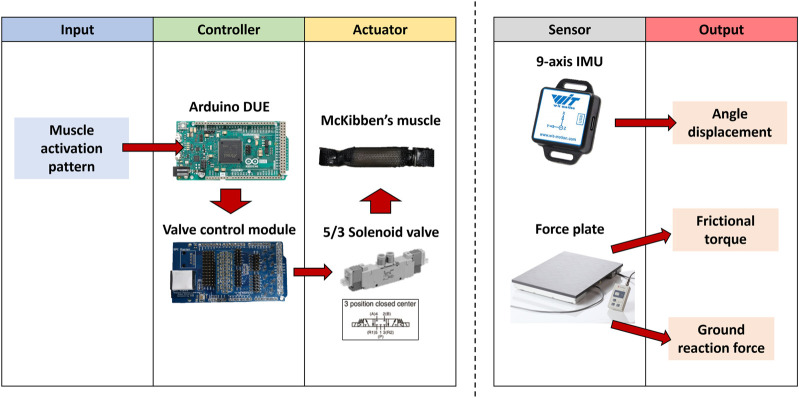
Control system of the musculoskeletal robot and data acquisition method.

**FIGURE 4:**
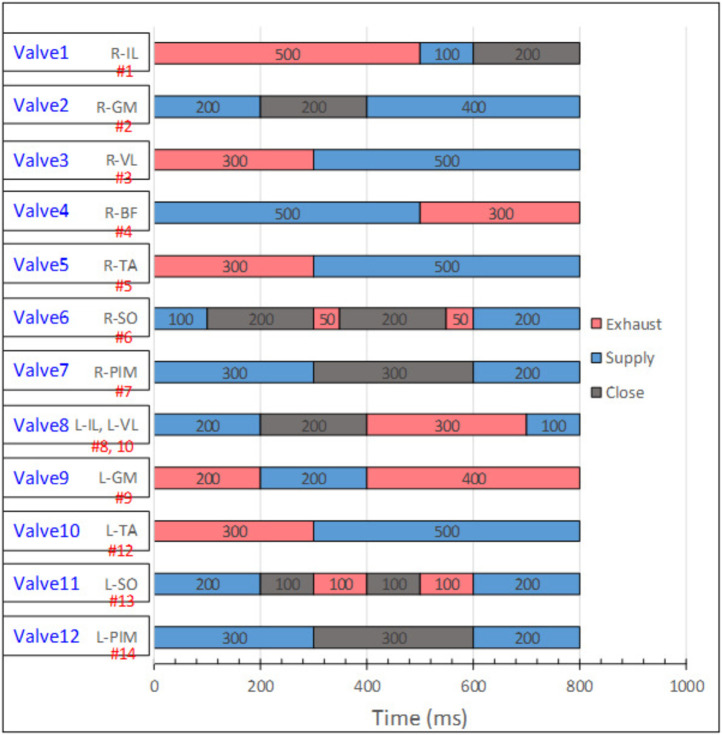
Muscle activation pattern for the slip-turning motion on the toe joint. Twelve valves were used for controlling all the 14 PAMs. Muscle names with R-XXX are the activations of the right leg, while L-XXX are the muscles on the left leg. The red portions indicate exhaustion of the PAMs; blue indicates supplying air to the PAMs, and the gray portions indicate the valve’s closing period.

The control system of the robot is depicted in Figure 3. To control the PAMs, a microcontroller (Arduino Due) connected to our laboratory valve control module was employed. The solenoid valves (VQZ1321-6L1-C6) used for controlling the muscles are of the five-port, three-position type, capable of supplying, exhausting, and closing the air opening. The musculoskeletal robot consists of 12 valves corresponding to 12 schematically connected muscles, as shown on the left side of Figure 4, as one solenoid valve is capable of controlling a single muscle activation pattern. The supplying air pressure used in this study is 0.6 MPa.

The slip-turning motion of the robot is achieved by providing a muscle activation pattern to the controller. The muscle activation pattern was created to actuate all 14 PAMs simultaneously in the correct sequence to realize the motion.

## 3 Slip-turning motion

### 3.1 Slip-turning strategy

The slip-turn was previously demonstrated by some motor-driven robots: WABIAN-2 (Hashimoto et al., 2011), HRP-4C (Miura et al., 2012), and others (Yeon and Park, 2014). Contrary to these robots, the slip-turning of our musculoskeletal robot refers to human biomechanics, where the slip-turning often occurs shortly for a small duration during walking. Among the aforementioned research, one research distinguishes the turning mechanics of humans into two types (Hase and Stein, 1999): step turn and spin turn. The spin turn strategy of these studies was used as the base model of our slip-turning motions for recreating the muscle activation pattern by studying the muscles’ activation timing with the electromyography signal (EMG) during the spin turn.

### 3.2 Muscle activation pattern

The control method for the bipedal robot is a joint control method that directly controls the angle of each joint through the activation of PAMs. To realize the slip-turning motion, the muscle activation pattern was specifically generated to replicate the joint movements observed during human spin turns. Each muscle was activated for a comparable duration and speed as indicated by the reference EMG pattern and joint angle measurements. The EMG pattern was used for generating the activation timing, as the signal provided explicit indications of muscle activation without any delay caused by signal transmission. In contrast to an electroencephalogram (EEG), the EMG pattern allowed for more accurate and immediate detection of muscle activation, making it a suitable choice for generating precise activation timings in the control process. By studying the EMG pattern, we can roughly estimate the activation time of the muscles. When the graph indicates muscle activation, the corresponding valve connected to the PAM represents that the muscle will undergo a change in its state. This change may involve supplying air, exhausting air, or closing the air supply, depending on the specific requirements of the muscle’s activation. Previous studies have highlighted that determining the timing of muscle activation alone does not provide sufficient information (Ogawa et al., 2011). Once the activation timing is established, additional adjustments need to be made through a process of trial and error to determine the appropriate valve states. This iterative approach allows for fine-tuning and optimization of the muscle activation patterns to achieve the desired performance and motion of the musculoskeletal robot.

**FIGURE 5:**
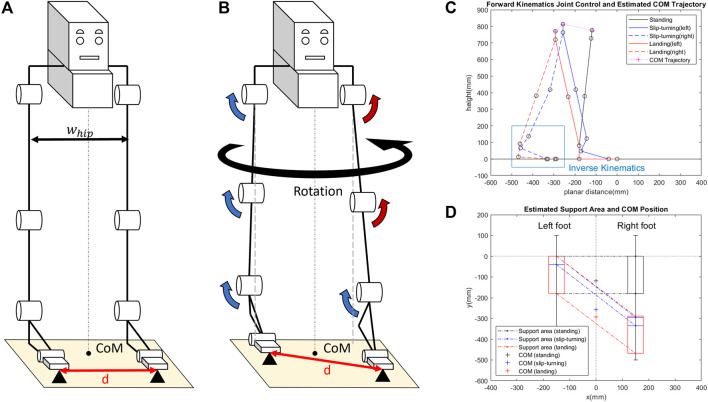
Slip-turning strategy based on the design from human spin turn. While the width of the hip *w*_*hip*_ stays the same before **(A)** and after **(B)** the motion, there is a small increase in the toe distance *d* from foot displacement after slip-turning. **(C)** Simulation of forward kinematics by the joint control method to find the estimated COM trajectory and foot placement. **(D)** Comparing the estimated support area to the COM position.

The activation time of the PAMs is controlled through the microcontroller, changing the valve state while causing some delays between each phase. The angular speed of each joint is a fixed value, manually adjusted by using flow control valves (SMC AS2002F-06) connected to the supplying air tube. In comparison to their experiments (Hase and Stein, 1999), our robot turns while standing still, instead of turning while walking, partials of the pattern were modified to adjust to our experiment. The total duration of the slip-turning pattern is 800 milliseconds. The completed muscle activation pattern used in this study is shown in Figure 4. Figures 5A, B depict the motion from standing to slip-turning.

Figure 5B describes the direction of each joint corresponding to the muscle activation pattern, noting that all joints and muscles behave similarly, as shown in the spin turn study. Figure 5C presents the simulation of the motion by using the joint control method. In the simulation, the forward kinematics method was used to calculate the joint positions, starting from the left toe and progressing upward to the left hip, toward the right hip, and then downward to the right ankle. Additionally, inverse kinematics was utilized to determine the placement of the right foot, pivoting around the toe joint, which demonstrates the foot’s adaptability and flexibility in response to different ground levels. The estimated COM trajectory is also shown in Figure 5C. The simulation was conducted with both hip joints set at the same height and without lateral swaying. The support polygon was calculated based on the foot position. Figure 5D illustrates the support polygon with the COM position, providing visual confirmation of the system to validate the equilibrium and stability of the system.

## 4 Experiments

**FIGURE 6:**
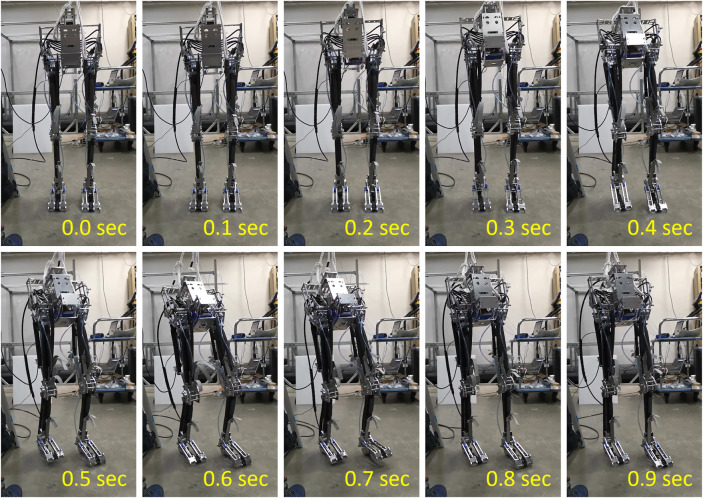
Realization of the slip-turning motion on toe joint of the musculoskeletal robot “PneuTurn‐T.”

The experiments were conducted to investigate the impact of utilizing toe joints and the PIM in the slip-turning motion. The realization of the slip-turning motion, implemented in our robot “PneuTurn‐T” using the muscle activation pattern, is shown in Figure 6. This motion was utilized consistently across all experiments, with slight variations in PIM activation based on the specific foot condition under investigation, allowing for data collection and analysis (see Figure 8).

### 4.1 Experimental setup

**FIGURE 7:**
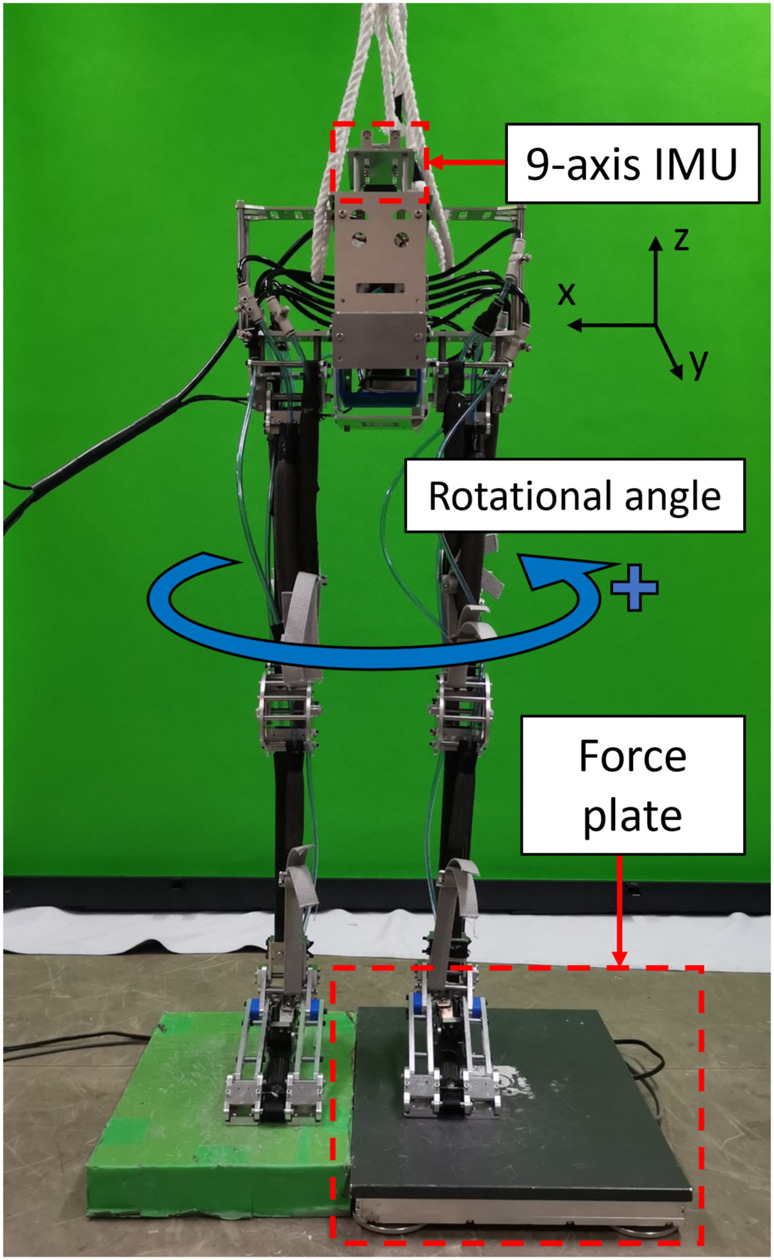
Experimental setup for collecting data with an IMU and a force plate under the left foot.

The experimental setup for data collection is illustrated in Figure 7. The robot was placed on a platform, with its left leg positioned on a force plate (TF-3040), for measuring the ground reaction force and frictional torque of the primary supporting foot. To monitor the robot’s rotational angle, a 9-axis IMU (BWT901CL) was installed on its body, with a positive angle indicating counterclockwise rotation as the robot turns to the left. Additionally, an IMU was affixed to the toe joint to measure the toe-lifting angle.

### 4.2 Different conditions of toe joints

**FIGURE 8:**
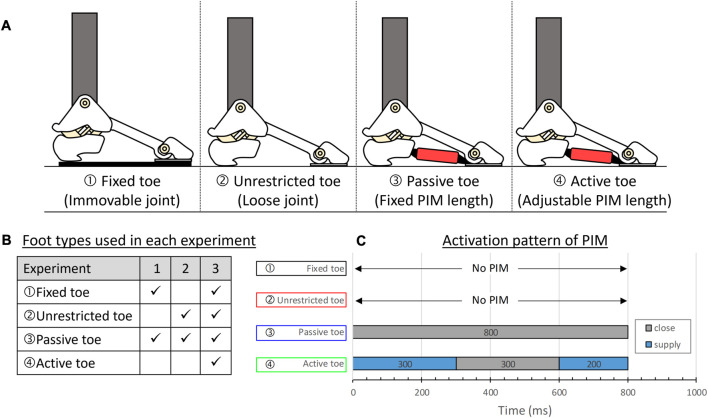
Different conditions of the toe used in experiments. **(A)** From the left: “Fixed toe” is a foot with a fixed toe joint acting as a rigid foot, “Unrestricted toe” is a foot with a loose toe joint without PIM, “Passive toe” is a foot with a fixed PIM length, and “Active toe” is a foot with an adjustable PIM length which is actuated at a certain moment. **(B)** Summary of foot conditions used for comparison in each experiment. **(C)** Activation pattern of the PIM.

The objective of the experiments was to investigate the effects of slip-turning on different types of feet. Four distinct foot configurations were utilized in the experiments, each contributing to the study as shown in Figure 8. The first type is a fixed toe foot, which emulates the behavior of a rigid foot or a foot without a toe joint. The second type is an unrestricted toe foot without PIM attachment, allowing the toe to move freely and lack stiffness. The third type is a passive toe foot with a movable toe connected to the PIM, where the length of the PIM is predetermined by the supplied volume with constant stiffness. The fourth type is an active toe foot, where the toe is actively actuated in response to the motion, generating slight movement in the toe joints.

The first experiment aimed to compare the turning behavior of a foot with a fixed toe to a foot with a passive toe, demonstrating the advantages of utilizing toe joints in slip-turning motion. In the second experiment, the toe-lifting angle was compared between a foot with an unrestricted toe and a foot with a passive toe, highlighting the role of foot stiffness provided by the PIM. Lastly, an experiment was conducted to compare the ground reaction forces among all foot types, investigating propulsion and postural stability during similar robot movements. The summary of all three experiments is shown in Figure 8B.

## 5 Results

### 5.1 Slip-turning in foot with and without the toe joint

**FIGURE 9:**
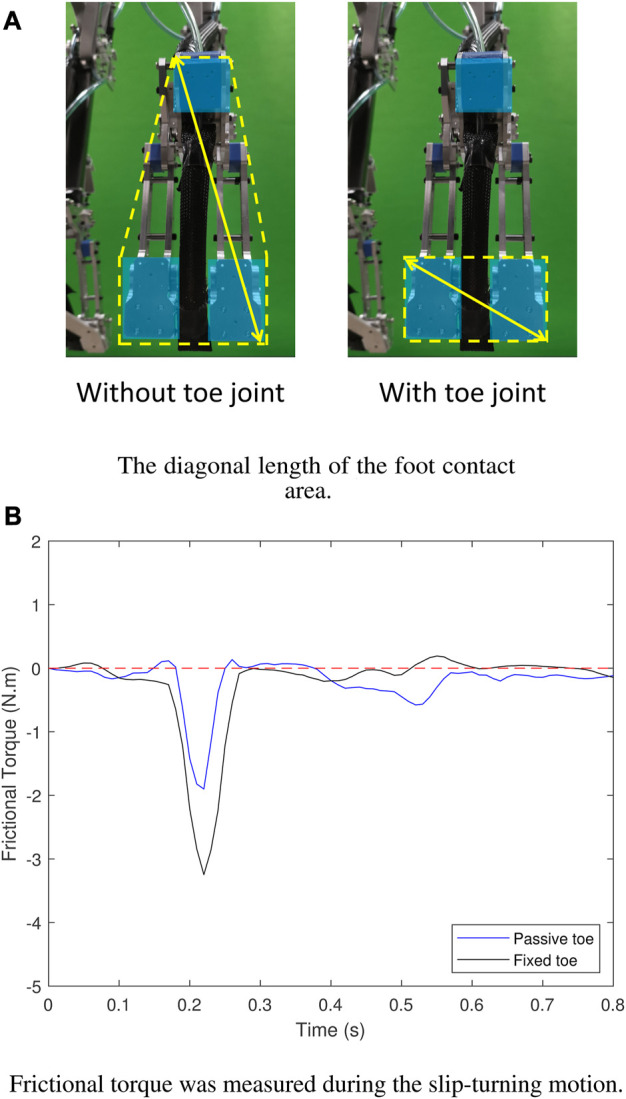
Results of frictional torque. **(A)** The diagonal length of the foot contact area was reduced by the utilization of the toe joint. **(B)** The frictional torque was reduced in the foot with toe joint (“Passive toe”) compared to the foot without the toe joint (“Fixed toe”).

In the first experiment, the contribution of using the toe joint in the slip-turning motion was examined by comparing the foot with a fixed toe to the foot with a passive toe. The results of frictional torque revealed a noticeable change in frictional torque at 0.2 s, as depicted in Figure 9B. The foot with the toe joint exhibited a smaller frictional torque during the motion, likely attributed to the reduction in the foot contact area and its diagonal length (Miura et al., 2012), as illustrated in Figure 9A.

**FIGURE 10:**
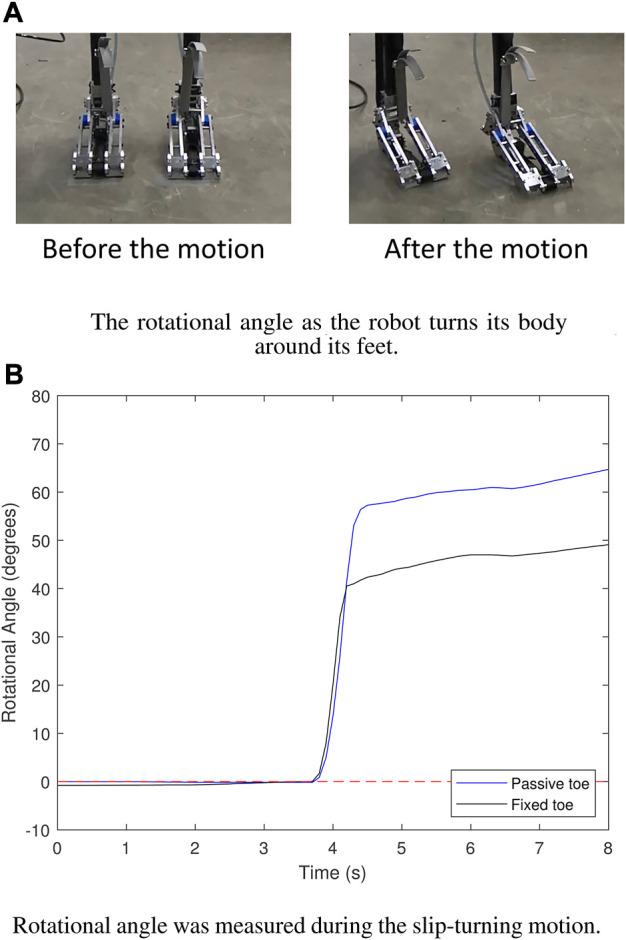
Results of the rotational angle. **(A)** The direction of the foot was changed along with its body alignment after the slip-turning motion. **(B)** The foot with “Passive toe” yielded a larger rotational angle than the foot with “Fixed toe.”

The second results show the rotational angle of the robot’s body measured by the 9-axis IMU installed on the robot’s body during slip-turning motion, as shown in Figure 10A. The results in Figure 10B demonstrate that the foot with a fixed toe achieved a rotational angle of approximately 50°, whereas the foot with a passive toe achieved a significantly improved rotational angle of up to 70°. This improvement suggests enhanced mobility, potentially resulting from the reduced frictional torque and increased flexibility of the foot with the passive toe joint (Ouezdou et al., 2005; Ogura et al., 2006; Hashimoto, 2020).

### 5.2 Foot stiffness and toe over-dorsiflexion

**FIGURE 11:**
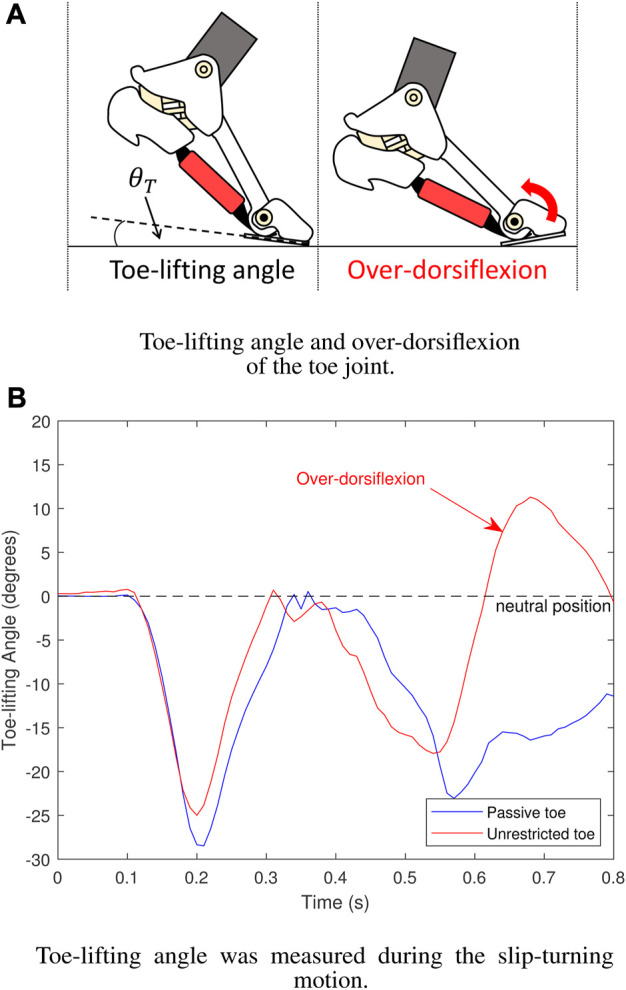
Results of toe-lifting angle. **(A)** Over-dorsiflexion of the toe. **(B)** The foot with a “Passive toe” prevents the toe joint from over-dorsiflexion compared to the foot with an “Unrestricted toe” without toe stiffness.

In the experiment comparing slip-turning on the foot with and without foot stiffness from the PIM, restraining the motion, the toe-lifting angle *θ*_*L*_ was measured to analyze the results. Figure 11A displays the toe-lifting angle, which was measured using an additional 9-axis IMU installed on the robot’s toe. Figure 11B indicates that the foot with an unrestricted toe or the foot without the PIM is more prone to experiencing toe over-dorsiflexion, while the stiffness provided by the PIM in the foot with a passive toe helps restrain and prevent over-dorsiflexion.

The occurrence of toe over-dorsiflexion can lead to a loss of postural balance in the robot due to reduced foot contact area in an unstable state, similar to individuals with floating toes (Fujimaki et al., 2021).

### 5.3 Propulsion and postural stability

**FIGURE 12:**
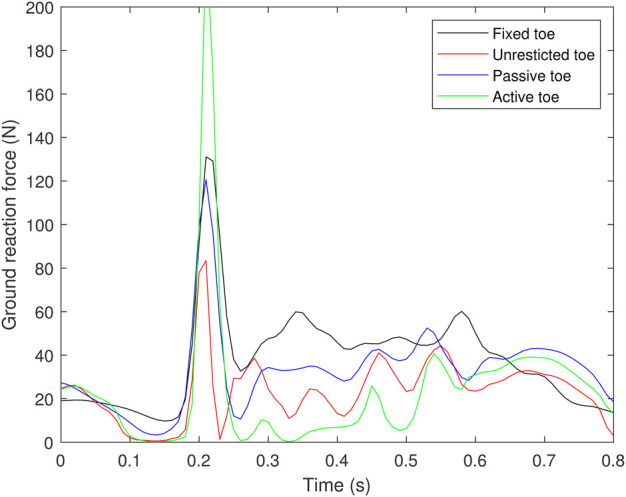
Results of ground reaction force.

The final experiment aimed to compare the ground reaction force (GRF) among all types of feet to assess the impact of the changing foot stiffness on the motion. The GRF pattern provided insights into the contributions of foot stiffness (see Figure 12). The results indicated that at the beginning of the motion, specifically at 0.2 s, the foot with an active toe exhibited the strongest propulsion among the four types of feet, followed by the fixed toe, passive toe, and unrestricted toe, respectively. The strong propulsion might indicate the capability of enhancing the motion of the toe joint with the PIM (Hughes et al., 1990; Agarwal and Popovic, 2018), transmitting the force from the upper leg to the phalanges, increasing its push-off force.

On the other hand, following the initial propulsion at 0.2 s, a noticeable decrease in ground reaction force (GRF) was observed in all types of feet, suggesting a potential disruption or an enhancement in postural stability during the robot’s motion. A higher GRF value indicated a greater weight transfer toward the left foot in the front, shifting the COM position closer to the anterior direction, which is favorable for maintaining the robot’s posture at the end of the motion.

## 6 Discussion

As our robot was designed with a human-like musculoskeletal structure, the position of the COM of the body was supposed to be placed in a similar manner. In humans, the support polygon aligns with the direction of the toes, pointing toward the anterior side of the body. During the neutral stance of a human, the COM tilts slightly forward in favor of balancing the whole-body stability, where the shifting of the COM is possible within the area of the support polygon. However, our robot, lacking an upper body, exhibits a posterior inclination of the COM, making it challenging to achieve a proper positioning of the COM for maintaining postural stability. Moreover, during rapid motions, although the foot structure aids in stabilizing the body, the absence of a balanced body weight amplifies the robot’s backward tendency, further compromising postural stability. Nonetheless, this characteristic of the robot could be utilized to further investigate the body stability in the absence of upper extremities, offering potential applications in medical practices for patients with upper-limb paralysis.

Due to the compressibility of air, the robots driven by the PAMs provide lower accuracy of joint control and unstable velocity (Chiang and Chen, 2017; Zhao and Song, 2020; Huang et al., 2022), which made them reluctant to achieve the task in the best manner constantly. Combined with the feedforward system of the robot, without any feedback control to improve the motion, and attempt to improve the motion solely by its structure and fine-tuning, making it even more difficult to remain in the right posture.

As previous studies have mentioned, the PIMs are more likely to contribute to foot stabilization than to balance control during postural challenges (Ku et al., 2012; Ferrari et al., 2020), such as body swaying, and the role of the PIM in this study focuses on the stabilization of the foot instead of attempting to control postural stability directly.

## 7 Conclusion

This paper demonstrated a turning motion of the musculoskeletal robot, which is usually unfavorable to execute due to its limited range of motion and complex controls. The bipedal robot could realize the slip-turning motion on toe joints about its yaw axis, while all eight joints of the robot were placed on the pitch axis. The foot with toe joints has enabled the heel-off motion of the musculoskeletal robot and could reduce the foot contact area during the turning motion compared to the rigid foot with a fixed toe, resulting in a reduction of frictional torque, minimizing its power, and an increase in the rotational angle. The second experiment proved that by using the PIM to restrain the toe joint, the robot could prevent the over-dorsiflexion of the toe, which can contribute to the improvement of static postural stability in the anterior–posterior direction. Meanwhile, the active toe could generate an even stronger propulsion, which can be useful in a quick motion. The unique structure of the human foot needs to be studied further to understand its contributions to the intrinsic tension force and the stability of the foot in a quick motion. Our findings contribute to the advancement of robotic systems that mimic biological structures and movements and widen the possibilities for future research in the field of robotics.

Future works include studies for optimizing control strategies for the PIM, exploring the adaptability of the foot, enhancing the motion, and integrating sensory feedback into the control system. These advancements will contribute to the development of more versatile and capable robots with improved locomotion abilities, enabling applications in various fields such as search and rescue, exploration, medical practice, and human-assistive robotics.

## Data Availability

The original contributions presented in the study are included in the article/Supplementary Material; further inquiries can be directed to the corresponding author.
